# Mediating Effects of Self-Efficacy, Benefits and Barriers on the Association between Peer and Parental Factors and Physical Activity among Adolescent Girls with a Lower Educational Level

**DOI:** 10.1371/journal.pone.0157216

**Published:** 2016-06-16

**Authors:** Maite Verloigne, Greet Cardon, Marieke De Craemer, Sara D’Haese, Ilse De Bourdeaudhuij

**Affiliations:** 1 Department of Movement and Sports Sciences, Faculty of Medicine and Health Sciences, Ghent University, Ghent, Belgium; 2 Research Foundation (FWO), Brussels, Belgium; Vanderbilt University, UNITED STATES

## Abstract

**Background:**

The prevalence of physical activity among lower educated adolescent girls is low, suggesting it is important to have insights into the complex processes that may underlie their physical activity levels. Therefore, this study aimed to examine the mediating effects of self-efficacy, perceived benefits and barriers on the associations between peer and parental variables and physical activity among lower educated adolescent girls.

**Methods:**

In total, 226 girls (mean age 16.0±1.0 years; 53% technical education; 47% vocational education) from a convenience sample of 6 secondary schools in Flanders, Belgium, completed a questionnaire on their total physical activity level and related peer and parental variables (i.e. modeling of physical activity, co-participation in physical activities and encouragement to be active) and personal variables (i.e. self-efficacy to be active, and specific perceived benefits of physical activity and specific barriers to be active). Mediating effects were tested using MacKinnon’s product-of-coefficients test based on multilevel linear regression analyses.

**Results:**

Higher peer and parental modeling, co-participation and encouragement were significantly related to a higher physical activity level among adolescent girls (p<0.05). Self-efficacy, the perceived benefits of having fun, being around friends or meeting new people, and not being bored and the perceived barrier of not liking physical activity mediated several associations between peer and parental variables and girls’ physical activity, with some of the mediated proportions exceeding 60%.

**Conclusions:**

This study contributed to a better understanding of the complexity of how parental and peer factors work together with personal factors to influence the physical activity levels of adolescent girls with a lower educational level. Interventions should involve both peers and parents, as they may influence girls’ physical activity both directly and indirectly through the internalisation of several personal variables, such as self-efficacy to be active and the perceived benefit of having fun.

## Introduction

Physical activity (PA) plays a key preventive role in the physical and mental health of children and adolescents [1,2]. The PA guidelines continue to underline participation in moderate-to-vigorous PA for 60 minutes per day [2,3], but many young people across Europe fail to achieve this recommendation [4]. A previous study has demonstrated that particularly adolescent girls have low levels of PA and that 90% of them do not reach the recommended 60 minutes per day [5]. Therefore, it has been advocated to target adolescent girls for interventions to promote PA [6,7], especially as this group has been found to be less interested in PA [8]. Moreover, adolescents with a lower educational level (i.e. attending technical/vocational education) are an important target group as well. This group engages in more unhealthy behaviours than counterparts with a higher educational level (i.e. attending general education) [9–11]. Therefore, efforts should be made to promote PA among this specific subgroup.

The first essential step to promote PA is to identify the factors that are associated with lower educated adolescent girls’ PA levels by applying and evaluating a theoretical approach [12]. The socio-ecological model [13] and the Environmental Research framework for weight Gain prevention or the EnRG framework [14] both integrate personal factors with environmental factors to understand why people perform (un)healthy behaviours. These models suggest that environmental factors (i.e. sociocultural, physical, economic and political environmental factors) can have a direct influence on adolescent girls’ behaviour via automatic pathways or an indirect influence via personal factors (i.e. mediators). Investigating the mediating effect of personal factors on how the environment is associated with girls’ PA levels enables an in-depth insight into the processes that may underlie lower educated adolescents girls’ behaviour which may lead to effective interventions.

Regarding the influence of environmental factors, it has been shown that particularly the social environment, and more specifically girls’ peers and parents, can have an important impact on girls’ PA levels. The influence of parents on adolescent girls’ PA level can occur through modeling and co-participation [15], but especially through supporting them, such as encouraging them or by providing logistic support [16–18]. Parents already play an important role in influencing girls’ health behaviour at a younger age [19], but the influence of peers may increase when girls enter adolescence [20]. It has even been suggested that peers or friends are more important than parents in influencing adolescent girls’ PA levels [21,22]. Moreover, adolescent girls with active friends are more likely to be active themselves and being physically active together with friends is associated with more PA [23].

Further, according to socio-ecological models [13,14], these associations between peer and parental factors and adolescent girls’ PA could possibly be mediated by personal factors, which means that peer and parental factors may influence girls’ PA indirectly through personal factors. This implies that these personal factors are also associated with adolescent girls’ PA. Important personal factors that are associated with PA are self-efficacy, perceived benefits of PA and perceived barriers to be physically active [24,25]. Self-efficacy has indeed been identified as one of the most important correlates of adolescent girls’ PA [26–29]: a higher feeling of self-efficacy, or beliefs in one’s capability to be physically active, was related to more PA among girls. Girls’ perception of benefits of PA (e.g. the benefit of being healthy and body image) has also shown to be positively associated with PA [29,30]. Finally, a higher perception of barriers to be active is associated with less PA among girls [29,31]. Examples of identified barriers related to less PA participation among girls are lack of time, inaccessibility of PA facilities and body-centered issues [32].

Although several studies have already investigated the direct relationship between these personal factors and adolescent girls’ PA, only few have specifically investigated the mediating effect of one or more of these variables on the association between social environmental factors and girls’ PA behaviour. However, it is important to investigate the more complex interactions involved in the mechanisms underlying behaviours such as PA, since health behaviours are likely to be complicated [14]. A more comprehensive understanding of how environmental and personal factors interact to affect PA is necessary for the development of effective interventions and health policies [33], which may be particularly important for lower educated adolescent girls. In a previous study conducted in US adolescent girls from the 6^th^ and 8^th^ grade, the perception of barriers mediated the association between parental social support and PA [34] and self-efficacy for overcoming barriers mediated the association between social support from significant others and PA [35]. Thus, there is a dearth of research exploring the mediating effect of personal factors on the association between social environmental variables and adolescent girls’ PA.

Therefore, the current study aimed to investigate the associations between peer and parental variables (i.e. modeling, co-participation and encouragement) and PA and the mediating effects of self-efficacy, perceived benefits and barriers on these associations in 10^th^ grade adolescent girls with a lower educational level from Flanders, Belgium. The first hypothesis was that both peer and parental variables would be associated with girls’ PA, but with stronger associations for peer than parental variables. The second hypothesis was that these associations would at least be partly mediated by self-efficacy, perceived benefits and perceived barriers.

## Methods

### Procedure

The cross-sectional data presented in this paper were drawn from the pre-test of an intervention study using a participatory approach to promote PA in Flemish adolescent girls. A convenience sample of six secondary schools providing vocational or technical education (East-Flanders, Flanders, Belgium) was selected and the schools agreed to participate in the study. All adolescent girls in the 10^th^ grade (including the majority of girls being 16 years old) of these schools were invited to participate in the pre-test data collection (in total 27 classes).

The study was explained by a researcher in the classroom; the girls were asked if they would voluntarily and anonymously complete a questionnaire on PA and possibly related personal, parental and peer factors in the classroom (S1 File). At the beginning of the questionnaire, the adolescent girls were informed that consent was automatically obtained when they voluntarily completed the questionnaire. It was also explained that not giving consent to participate would not result in any consequences for the girls. Additional informed consent of parents or guardians was not obtained since parental consent is not required for participants from middle to late adolescence for questionnaires when questions are not related to a sensitive topic [36,37] and when information is related to a non-identified or non-identifiable natural person [38]. This study protocol was approved by the ethics committee of the Ghent University Hospital (B670201420580) referring to The Privacy Act of December 8th, 2012 on the protection of privacy in relation to the processing of personal data [38]. An online or paper and pencil version of the questionnaire could be used, depending on the preference of the school. The explanation of the study in combination with filling in the questionnaire was scheduled to be executed in one lesson time (about 50 minutes). The questionnaire took about 20 minutes to complete. In total, 226 girls were included in the analyses (response rate: 88%; S2 File). Pre-test data collection occurred on schooldays in September and October 2014.

### Measures

#### Peer and parental variables

Three peer and three parental variables were assessed in the questionnaire using questions derived from previous studies examining health behaviour in adolescents [39–41]. Variables were peer and parental modeling, co-participation and encouragement (Table 1) and were measured on a five-point Likert scale, ranging from ‘never’ to ‘always’.

**Table 1:** Description of peer, parental and personal variables.

Variable	Questionnaire item	Response category
**Peer variables**		
Peer modeling	How often are your friends physically active?	1 = Never, 2 = Almost never, 3 = Sometimes, 4 = Often, 5 = Always
Peer co-participation	How often are your friends physically active together with you?	
Peer encouragement	How often do your friends encourage you to be physically active?	
**Parental variables**		
Parental modeling	How often are your parents physically active?	1 = Never, 2 = Almost never, 3 = Sometimes, 4 = Often, 5 = Always
Parental co-participation	How often are your parents physically active together with you?	
Parental encouragement	How often do your parents encourage you to be physically active?	
**Personal variables**		
Self-efficacy to be physically active (mean value of four items with Cronbach’s alpha = 0.85)	I could be active even (1) if I have to get up early; (2) if my friends want to do something else; (3) if I have a lot of work for school; (4) if it is hard or difficult	1 = Strongly disagree, 2 = Partly disagree, 3 = Neither agree nor disagree, 4 = Partly agree, 5 = Strongly agree
Perceived benefit of health	I think physical activity is good because it improves my aerobic condition and health	
Perceived benefit of being around friends or meeting new people	I think physical activity is good because I am with friends or I meet new people	
Perceived benefit of fun	I think physical activity is good because I get fun out of physical activities	
Perceived benefit of being better than others	I think physical activity is good because during physical activities, I can show that I am better than others	
Perceived benefit of not feeling bored	I think physical activity is good because otherwise I would feel bored	
Perceived benefit of body image	I think physical activity is good because I lose weight and improve my body	
Perceived barrier of lack of time	I am not able to participate in physical activities because I do not have enough time	
Perceived barrier of not liking PA	I am not able to participate in physical activities because I do not like it	
Perceived barrier of not being good at PA	I am not able to participate in physical activities because I am not good at it	
Perceived barrier of lack of transportation to physical activities	I am not able to participate in physical activities because I do not have transportation to get there	
Perceived barrier of not being allowed to do physical activities	I am not able to participate in physical activities because my parents do not allow me	

PA, physical activity

#### Personal variables

The questionnaire also assessed a range of personal variables (Table 1), derived from previous studies among adolescents [39–41] and based on a reliable and validated questionnaire for measuring determinants of PA [42,43]. The mean value of four items was used to measure girls’ self-efficacy to be physically active (Cronbach alpha = 0.85). The questionnaire also assessed six possible benefits of physical activity and five possible barriers to be physically active. Exploratory factor analyses revealed no factors and in order to provide the most accurate information on the possible underlying mediating mechanisms, it was decided to use the single items for benefits and barriers as mediators in the analyses. All personal variables were measured on a five-point scale, ranging from ‘strongly disagree’ to ‘strongly agree’. For all variables, a higher mean value represents a higher form of the variable.

#### Total physical activity level

Girls’ total PA level was determined using the Flemish Physical Activity Questionnaire, which has been found reliable and valid among adolescents [44]. The Flemish Physical Activity Questionnaire assesses different domains of PA in order to calculate the total PA level of a person. Active transportation (i.e. minutes spent in active transportation to school and in leisure time) and sport participation (i.e. minutes spent in physical education and extra-curricular physical activities at school and in physical activities during leisure time) were summed to provide an estimate of adolescent girls’ total PA. For the purpose of this paper, only total PA was used as an outcome.

### Statistical analyses

Linear regression analyses were conducted using SPSS version 22.0 (SPSS Inc., Chicago, IL, USA). Clustering at the school level was taken into account by conducting multi-level analyses. The Likelihood-Ratio-Test showed that the model with school level was a significant better model than the single model (chi² = 407.46; p<0.001). Total PA was checked for normality first and was normally distributed (skewness of 0.69 and kurtosis of -0.05). Girls’ age was included as a covariate. The mediation analyses consisted of the following steps. Firstly, main associations between each parental or peer variable and adolescent girls’ daily total PA were examined (τ-coefficient). In the second stage, the mediating role of self-efficacy, and the different benefits and barriers was examined using the product-of-coefficients test of MacKinnon et al. [45]. This test includes the following steps: (1) estimation of the associations between each parental/peer variable and potential mediators (Action Theory test; α-coefficients or a-path); (2) estimation of the associations between the potential mediators and girls’ total PA level (Conceptual Theory Test; β-coefficients or b-path), adjusting for the relevant parental/peer variable; and (3) calculation of the product-of-coefficients (αβ), representing the mediated effect. Statistical significance of the mediated effect was estimated by dividing αβ by its standard error (SE). To calculate SE, the Sobel test was used: SE (αβ) = √(α2*SE (β)2 + β2*SE (α)2) [46]. The percentage mediating the association between parental/peer variables and girls’ total PA level was calculated by dividing αβ by the τ-coefficient. Finally, after assessing single mediating models, multiple mediating models were assessed including only those variables that were found to be significant mediators in single mediating models. The total multiple mediation was investigated through a model including all significant single mediators simultaneously. The significance level was set at 0.05.

## Results

### Descriptive statistics

Two hundred and twenty six adolescent girls participated in the study with a mean age of 16.02 ± 0.98 years. All girls had a lower educational level: 53% followed technical education and 47% vocational education. The mean values of the peer/parental variables, personal variables and total PA levels are presented in Table 2.

**Table 2:** Sample characteristics and descriptive statistics.

Demographic variables	mean ± SD or percentages
Age (years)	16.02 ± 0.98
Technical education	53.3%
Vocational education	46.7%
**Physical activity variables**	**mean ± SD**
Total physical activity (mins/day)	55.83 ± 31.83
**Peer variables**	**mean ± SD**
Peer modeling (range 1–5)	2.90 ± 0.92
Peer co-participation (range 1–5)	2.47 ± 1.13
Peer encouragement (range 1–5)	2.29 ± 1.26
**Parental variables**	**mean ± SD**
Parental modeling (range 1–5)	2.55 ± 1.13
Parental co-participation (range 1–5)	1.79 ± 0.95
Parental encouragement (range 1–5)	2.85 ± 1.35
**Personal variables**	**mean ± SD**
Self-efficacy to be physically active (range 1–5)	3.06 ± 1.22
Perceived benefit of health (range 1–5)	4.45 ± 0.90
Perceived benefit of being around friends or meeting new people (range 1–5)	3.76 ± 1.19
Perceived benefit of fun (range 1–5)	3.82 ± 1.22
Perceived benefit of being better than others (range 1–5)	2.33 ± 1.38
Perceived benefit of not feeling bored (range 1–5)	3.60 ± 1.34
Perceived benefit of body image (range 1–5)	4.03 ± 1.25
Perceived barrier of lack of time (range 1–5)	2.99 ± 1.43
Perceived barrier of not liking PA (range 1–5)	2.59 ± 1.54
Perceived barrier of not being good at PA (range 1–5)	2.39 ± 1.43
Perceived barrier of lack of transportation to physical activities (range 1–5)	2.55 ± 1.43
Perceived barrier of not being allowed to do physical activities (range 1–5)	1.81 ± 1.27

SD, standard error; mins, minutes; PA, physical activity

### Associations between peer and parental variables and total physical activity (τ-coefficient)

Main associations between peer and parental variables and total PA are presented in Table 3. All peer and parental variables (i.e. modeling, co-participation and encouragement) were significantly positively associated with adolescent girls’ total PA levels. The τ-coefficients show the magnitude of the significant associations between peer and parental variables and total PA. For example, for each unit increase in peer modeling of PA, girls’ total PA level per day will increase by 10.45 minutes.

**Table 3:** Main associations between peer/parental variables and girls’ total PA level.

	Total PA level (τ (SE))
**Peer variables**	
Peer modeling of PA	10.45 (2.28)***
Peer co-participation in PA	9.34 (1.74)***
Peer encouragement to be physically active	7.72 (1.57)***
**Parental variables**	
Parental modeling of PA	5.24 (1.79)**
Parental co-participation in PA	9.61 (2.10)***
Parental encouragement to be physically active	5.95 (1.49)***

PA, physical activity; SE, standard error

** p<0.01

*** p<0.001

### Associations between peer and parental variables and potential mediators (α-coefficients)

All peer and parental variables were significantly positively associated with the perceived benefits of having fun and not being bored (Table 4). All peer and parental variables–except parental encouragement—were significantly positively associated with self-efficacy (Table 4). All peer variables and parental modeling were also significantly positively associated with girls’ perceived benefit of being around friends or meeting new people. Peer and parental encouragement to be physically active were significantly positively associated with girls’ perceived benefit of losing weight or improving their body. Peer modeling, peer and parental co-participation and parental encouragement were significantly inversely associated with the perceived barrier of not liking PA. Peer and parental co-participation were significantly inversely related to the perceived barrier of not being good in PA. The α-coefficients show the magnitude of the significant associations between peer and parental variables and potential mediators. For example, for each unit increase in peer modeling of PA, there is an increase of 0.54 for self-efficacy.

**Table 4:** Mediation analyses for associations between peer/parental variables and girls’ total PA level.

Total PA level				
	α (SE)	β (SE)	αβ (SE)	% mediated
**Peer modeling of PA**				
Self-efficacy	0.54 (0.09)***	9.94 (1.71)***	5.37 (1.29)***	51.6%
Benefit health	0.27 (0.07)***	4.92 (2.31)*	1.33 (0.71)	-
Benefit friends/new people	0.39 (0.09)***	6.61 (1.73)***	2.58 (0.90)**	24.8%
Benefit fun	0.49 (0.09)***	7.00 (1.70)***	3.43 (1.05)**	33.0%
Benefit better than others	0.15 (0.10)	3.73 (1.48)*	0.56 (0.43)	-
Benefit not bored	0.52 (0.09)***	4.29 (1.59)**	2.23 (0.91)*	21.2%
Benefit weight and body	0.18 (0.10)	3.39 (1.59)*	0.61 (0.44)	-
Barrier time	-0.11 (0.11)	-0.73 (1.43)	0.08 (0.18)	-
Barrier not liking	-0.55 (0.11)***	-2.79 (1.38)*	1.54 (0.82)	-
Barrier not good at it	-0.21 (0.11)	-2.55 (1.45)	0.54 (0.41)	-
Barrier no transportation	-0.07 (0.11)	1.03 (1.42)	-0.07 (0.15)	-
Barrier not allowed	-0.01 (0.10)	1.88 (1.63)	-0.02 (0.19)	-
All significant mediators together	**-**	**-**	6.50 (1.39)***	62.4%
**Peer co-participation**				
Self-efficacy	0.38 (0.07)***	9.51 (1.66)***	3.61 (0.92)***	38.6%
Benefit health	0.11 (0.05)**	6.16 (2.18)**	0.68 (0.39)	-
Benefit friends/new people	0.35 (0.07)***	6.20 (1.73)***	2.17 (0.75)**	23.2%
Benefit fun	0.40 (0.07)***	6.54 (1.70)***	2.62 (0.82)**	28.0%
Benefit better than others	0.14 (0.08)	3.99 (1.45)**	056 (0.38)	-
Benefit not bored	0.44 (0.07)***	4.09 (1.57)**	1.80 (0.75)*	18.9%
Benefit weight and body	0.12 (0.07)	3.82 (1.55)*	0.46 (0.33)	-
Barrier time	-0.11 (0.08)	-1.06 (1.38)	0.12 (0.17)	-
Barrier not liking	-0.33 (0.09)***	-3.41 (1.31)**	1.13 (0.53)*	12.0%
Barrier not good at it	-0.22 (0.08)**	-2.54 (1.43)	0.56 (0.38)	-
Barrier no transportation	0.06 (0.08)	-0.06 (1.37)	-0.004 (0.08)	-
Barrier not allowed	-0.001 (0.72)	1.99 (1.61)	-0.002 (1.43)	-
All significant mediators together	**-**	**-**	4.12 (1.17)***	43.7%
**Peer encouragement of PA**				
Self-efficacy	0.18 (0.06)**	10.27 (1.56)***	1.85 (0.68)**	23.9%
Benefit health	0.04 (0.05)	6.94 (2.17)**	0.28 (0.36)	-
Benefit friends/new people	0.13 (0.06)*	7.54 (1.63)***	0.98 (0.50)	-
Benefit fun	0.13 (0.06)*	7.97 (1.57)***	1.04 (0.52)*	13.3%
Benefit better than others	0.04 (0.07)	4.61 (1.45)**	0.18 (0.33)	-
Benefit not bored	0.21 (0.07)**	5.30 (1.48)***	1.11 (0.48)*	14.4%
Benefit weight and body	0.21 (0.07)**	3.13 (1.60)	0.66 (0.40)	-
Barrier time	-0.12 (0.08)	-1.10 (1.39)	0.13 (0.19)	-
Barrier not liking	-0.14 (0.08)	-4.19 (1.28)**	0.59 (0.38)	-
Barrier not good at it	-0.08 (0.07)	-3.25 (1.42)*	0.26 (0.25)	-
Barrier no transportation	-0.02 (0.08)	0.36 (1.38)	-0.01 (0.04)	-
Barrier not allowed	0.003 (0.06)	1.91 (1.63)	0.01 (0.12)	-
All significant mediators together	**-**	**-**	1.93 (0.68)**	25.0%
**Parental modeling of PA**				
Self-efficacy	0.26 (0.07)***	10.91 (1.64)***	2.84 (0.88)**	54.6%
Benefit health	0.10 (0.05)*	6.74 (2.27)**	0.67 (2.27)	-
Benefit friends/new people	0.16 (0.07)*	7.90 (1.69)***	1.26 (0.62)*	24.1%
Benefit fun	0.16 (0.07)*	8.30 (1.62)***	1.33 (0.64)*	25.7%
Benefit better than others	0.23 (0.08)**	4.16 (1.54)**	0.96 (0.49)*	18.6%
Benefit not bored	0.22 (0.08)**	5.87 (1.53)***	1.29 (0.58)*	25.1%
Benefit weight and body	0.06 (0.07)	4.35 (1.61)**	0.26 (0.32)	-
Barrier time	-0.19 (0.08)**	-1.22 (1.45)	0.23 (0.29)	-
Barrier not liking	-0.15 (0.09)	-4.50 (1.32)***	0.68 (0.45)	-
Barrier not good at it	-0.15 (0.08)	-3.29 (1.47)*	0.49 (0.34)	-
Barrier no transportation	-0.07 (0.08)	0.45 (1.43)	-0.03 (0.11)	-
Barrier not allowed	0.12 (0.07)	1.40 (1.70)	1.72 (2.09)	-
All significant mediators together	**-**	**-**	3.17 (0.94)***	61.7%
**Parental co-participation in PA**				
Self-efficacy	0.33 (0.08)***	10.14 (1.62)***	3.35 (0.97)***	34.9%
Benefit health	0.07 (0.06)	6.73 (2.19)**	0.47 (0.43)	-
Benefit friends/new people	0.11 (0.08)	7.85 (1.62)***	0.86 (0.65)	-
Benefit fun	0.27 (0.08)***	7.62 (1.61)***	2.06 (0.75)**	21.4%
Benefit better than others	0.20 (0.09)*	4.03 (1.48)**	0.81 (0.47)	-
Benefit not bored	0.28 (0.09)**	5.35 (1.49)***	1.50 (0.64)*	15.5%
Benefit weight and body	0.07 (0.09)	4.22 (1.56)**	0.30 (0.40)	-
Barrier time	-0.07 (0.10)	-1.60 (1.39)	0.11 (0.19)	-
Barrier not liking	-0.30 (0.11)**	-3.88 (1.31)**	1.16 (0.58)*	12.1%
Barrier not good at it	-0.28 (0.10)**	-2.60 (1.46)	0.73 (0.48)	-
Barrier no transportation	0.16 (0.10)	-0.42 (1.39)	-0.07 (0.23)	-
Barrier not allowed	0.10 (0.09)	1.32 (1.65)	0.13 (0.20)	-
All significant mediators together	**-**	**-**	4.18 (0.95)***	42.7%
**Parental encouragement of PA**				
Self-efficacy	0.11 (0.06)	10.80 (1.56)***	1.19 (0.67)	-
Benefit health	0.06 (0.04)	6.89 (2.21)**	0.41 (0.31)	-
Benefit friends/new people	0.10 (0.06)	7.83 (1.65)***	0.78 (0.50)	-
Benefit fun	0.05 (0.06)	8.55 (1.57)***	0.43 (0.52)	
Benefit better than others	0.05 (0.07)	4.62 (1.48)**	0.23 (0.33)	-
Benefit not bored	0.07 (0.07)	6.10 (1.47)***	0.43 (0.44)	-
Benefit weight and body	0.27 (0.06)***	3.01 (1.66)	0.81 (0.48)	-
Barrier time	-0.07 (0.07)	-1.46 (1.41)	0.10 (0.14)	-
Barrier not liking	-0.20 (0.07)**	-4.09 (1.32)**	0.82 (0.39)*	13.7%
Barrier not good at it	-0.12 (0.07)	-3.15 (1.45)*	0.38 (0.28)	-
Barrier no transportation	-0.05 (0.07)	0.45 (1.40)	-0.02 (0.08)	-
Barrier not allowed	-0.13 (0.06)*	3.04 (1.67)	-0.40 (0.28)	-

PA, physical activity

* p<0.05

** p<0.01

*** p<0.001

### Associations between potential mediators and total physical activity (β-coefficients)

In all single mediation models, the β-path or the conceptual theory test that investigates the association between the personal variables and girls’ total PA, was significant and positive for self-efficacy, perceived benefits of improving health, being around friends or meeting new people, having fun, being better than others and not being bored, and significant and negative for the perceived barrier of not liking PA (Table 4). In four out of six models, the β-path was significant and positive for the perceived benefit of losing weight or improving their body. In two out of six models, the β-path was significant and negative for the perceived barrier of not being good at PA. The β-path was not significant for the perceived barriers of lack of time, not having transportation, and not being allowed to do physical activities. The β -coefficients show the magnitude of the significant associations between the potential mediators and girls’ total PA level. For example in the first model, for each unit increase in self-efficacy, girls’ total PA level per day will increase by 9.94 minutes.

### Mediating effects of personal variables on the associations between peer and parental variables and total physical activity (αβ-coefficients)

**Fig 1:**
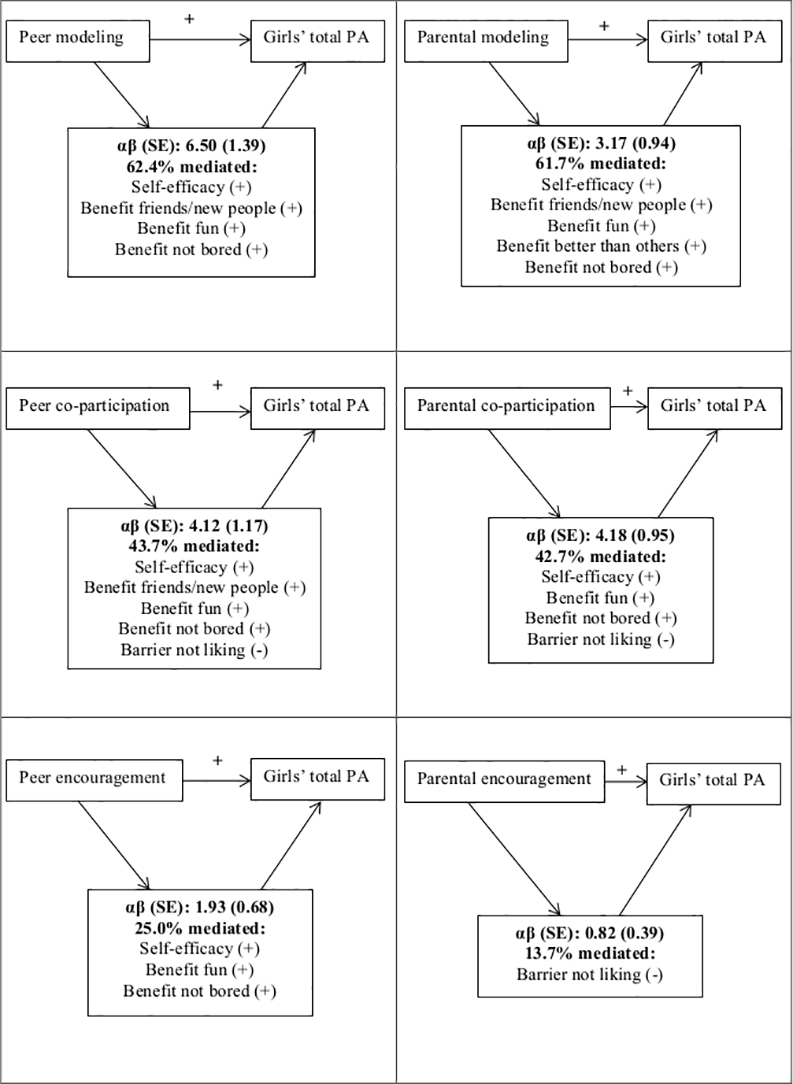
Visual presentation of the significant mediators on the association between peer and parental variables and girls’ total physical activity.

The results of the single mediation models are presented in Table 4. Self-efficacy, and the perceived benefits of having fun and not being bored mediated all associations between peer/parental variables and girls’ total PA, except for the association with parental encouragement. The perceived benefit of being around friends or meeting new people mediated the associations between all peer variables and girls’ total PA and between parental modeling and girl’s total PA. The perceived barrier of not liking PA also mediated the association between peer and parental co-participation and girls’ PA. Moreover, it was the only variable that mediated the association between parental encouragement and girls’ total PA. The perceived benefit of being better than others also mediated the association between parental modeling and girls’ total PA. The proportion mediated by the combination of all significant single mediators was 62.4% for peer modeling, 43.7% for peer co-participation, 25.0% for peer encouragement, 61.7% for parental modeling, and 42.7% for parental co-participation. As there was only one variable that significantly mediated the association between parental encouragement and girls’ total PA, no multiple mediation model was created. Fig 1 provides a visual presentation of the mediating effects of personal variables on the association between all peer and parental variables and adolescent girls’ total PA level.

## Discussion

Results of the present study showed that all peer and parental factors (i.e. modeling, co-participation and encouragement) were positively associated with the total PA level of adolescent girls with a lower educational level. Moreover, these positive associations were mediated by several personal variables, such as girls’ self-efficacy to be physically active, and the perceived benefits of having fun, being around friends or meeting new people, and not being bored. The proportion of the associations between the peer and parental variables mediated by personal factors was generally high, with some of the proportions exceeding 60%.

In previous research, the influence of peers was an important factor to explain girls’ PA during adolescence [21,22]. This finding is confirmed by the present study for lower educated girls since peer modeling, peer co-participation and peer encouragement were all positively related to girls’ total PA. Per one unit increase in the peer variables, there is an increase of 7 to 10 minutes total PA per day. This suggests that it is important to emphasise the social aspect within PA initiatives for adolescent girls [20,22] by focusing on doing physical activities with friends and promoting the message to encourage each other to participate in these activities [23]. Another possibility to involve the peer aspect in PA programmes is by using peer-tutoring as an intervention component [47]. The school setting might be considered as an appropriate setting to organise PA initiatives for girls [47]. Further, the importance of parental influences on PA levels of adolescent girls with a lower educational level should not be underestimated. It is often believed that parents are only or mostly influential at a younger age, but our results showed that parental modeling, co-participation and encouragement were also positively associated with girls’ total PA, although the magnitude of associations was somewhat lower for the parental variables. These results are in line with the first hypothesis of this study that both peer and parental variables would be associated with girls’ PA, but with (somewhat) stronger associations for peer than parental variables. Per one unit increase in the parental variables, there is an increase of 5 to 9 minutes total PA per day. Other studies confirmed that especially parental support (such as encouraging them to be active) is important for girls [48,49], although another study showed that parental encouragement was inversely related to adolescent girls’ PA probably because the encouragement was perceived as forced or overbearing by girls [50]. Nevertheless, the findings implicate that parents should be made aware of their continuous role in influencing their daughters’ PA levels. Future interventions could include strategies for parents to adopt an active lifestyle themselves, to do physical activities together with their daughter, and to encourage their daughters to be active. There have already been effective mother-daughter interventions to increase physical activity and/or to improve fitness that focused on doing activities together and supporting each other [51–53], so this might work for lower educated adolescent girls as well. In sum, an intervention that focuses on promoting PA among lower educated adolescent girls should target the social environment. The finding that both peers and parents were important could be explained by the lower educational level of the adolescent girls. It has been found that this subgroup relies or depends more on their social environment (including parents and peers) than their higher educated counterparts [54], which could be reflected in a higher influence of peers and parents on their total PA level. However, more research is needed to confirm this hypothesis.

Several personal factors mediated the associations between these peer and parental factors and girls’ total PA. This is in accordance with the socio-ecological model for PA [13] and the EnRG-framework [14] stating that environmental factors can have a direct influence on PA behaviour or an indirect influence via personal factors. Girls’ self-efficacy to be physically active and their perceived benefits of having fun and not being bored were important intermediate factors, as they mediated all positive associations between peer and parental variables and girls’ total PA, except for the association with parental encouragement. Self-efficacy has previously been identified as a mediator among adolescent girls, although only on the positive association between social support from significant others and PA [35]. Thus, the current study adds that next to peer and parental encouragement (which may be considered as a form of social support), peer and parental modeling and co-participation may also positively influence girls’ PA level indirectly through a higher feeling of self-efficacy. Girls’ perceived benefit of being around friends or meeting new people also mediated the positive associations between all peer variables and girls’ total PA and between parental modeling and girl’s total PA. Our results suggest that if adolescent girls have active peers and parents, do physical activities together with peers and receive encouragement from their peers to be active, they may perceive more benefits related to PA (or in others words, have positive cognitions towards PA), resulting in higher PA levels. In conclusion, peers and parents have the ability to foster adolescent girls’ positive cognitions towards PA. Moreover, it was notable that peers and parents positively influenced adolescent girls’ PA by increasing the perceived benefits that were related to fun and friends and not related to improving body and weight. We consider the fact that girls were physically active because of internal or autonomous motives, such as having fun and being around friends, as a positive result [48], as this is related to participation in PA on the long-term [55]. Peers and parents also positively influenced girls’ total PA by increasing the perceived benefit of not being bored. It might be possible that the peers and parents positively influenced the notion that doing physical activities is a useful way of spending leisure-time, which may result in more PA among lower educated girls.

Girls’ perceived barriers to be physically active were less important as mediating factors, suggesting that peers and parents do not influence adolescent girls’ PA through decreasing their perception of barriers to be physically active. This is not in line with the study of Dishman et al. [34] who found that perceived barriers mediated the positive association between parental support and adolescent girls’ PA. However, our study focused specifically on adolescent girls with a lower educational level. It could be that the positive influence of parental support on PA does not occur through a decrease in perceived barriers for this specific subgroup. Another important difference is that we chose not to compose a single sum score to assess girls’ perceived barriers, but to analyse every barrier separately in order to obtain more specific information on the nature of the barrier. In this study, only the perceived barrier of not liking PA was identified as a mediator in the positive association with peer and parental co-participation and parental encouragement. It was the only perceived barrier that was consistently inversely associated with girls’ total PA. This perceived barrier has indeed been identified as one of the main barriers among adolescent girls in previous studies [56,57], although it was expected that the perceived barrier of lack of time would also be significantly inversely associated with girls’ total PA [32,57]. A possible explanation could lay in our specific sample of girls with a lower educational level, as these girls may spend less time doing schoolwork and therefore have quite some leisure time. This would also explain the positive association between the perceived benefit of not being bored and girls’ total PA.

The positive association between parental encouragement and girls’ total PA was only mediated by one personal variable, i.e. the perceived barrier of not liking PA. The proportion mediated was quite low (14%). This may suggest that other personal variables could mediate this specific association, or that parental encouragement has a direct or automatic influence on girls’ total PA.

A first important study strength is the investigation of the perceived benefits and barriers separately as possible mediators, instead of calculating a sum score. This allows us to provide the most accurate information on the processes that may underlie adolescents girls’ PA behaviour. Another strength is the focus on adolescent girls with a lower educational level, as they are an important at-risk group regarding PA. There are also some study limitations that need to be acknowledged. First, cross-sectional data were used, suggesting that longitudinal or experimental study designs are needed to confirm causal associations between peer and parental variables, personal variables and adolescent girls’ PA. Another limitation is the use of questionnaires to assess girls’ total PA level. The Flemish Physical Activity Questionnaire has proven to be sufficiently reliable and valid [44], but recall bias and social desirability could play a role in the accuracy of the responses. Using objective measurements could be recommended, but the main problem is that adolescents have the lowest accelerometer wearing compliance of all age groups [58], which might result in a lower sample size. Another limitation is that we only explored associations with adolescent girls’ total PA level. It could be relevant to look at specific domains of PA as well, as distinct domains of PA have different associated factors [59], but it is of importance that the associated factors would then also be domain-specific. A final limitation is that we did not distinguish between the specific influence of the mother or father on adolescent girls’ PA, as our questionnaire only contained questions on parents in general.

## Conclusions

Both peers and parents play an important role in influencing adolescent girls’ with a lower educational level through modeling, co-participation and encouragement. The mechanism of peer and parental influences on adolescent girls’ PA occurs (at least partly) indirectly by fostering girls’ self-efficacy to be active and the perceived benefits of having fun, being around friends or meeting new people, and not being bored and by decreasing the perceived barrier of not liking PA. This suggests that these personal factors could also be affected in a PA-promoting intervention for lower educated adolescent girls by involving both parents and peers. Our study findings should be verified in longitudinal and experimental research studies.

## Supporting Information

S1 File Questionnaire for adolescent girls in Dutch. (DOCX)

S2 File Database mediating effects of self-efficacy, benefits and barriers on the association between peer and parental factors and physical activity among adolescent girls with a lower educational level. (XLSX)
