# Supplementary material for: Mediating Effects of Self-Efficacy, Benefits and Barriers on the Association between Peer and Parental Factors and Physical Activity among Adolescent Girls with a Lower Educational Level
Source: PLoS One. 2016 Jun 16;11(6):e0157216. doi: 10.1371/journal.pone.0157216 (PMC4911140; doi:10.1371/journal.pone.0157216)
Supplement: S1 File — (DOCX) [file pone.0157216.s001.docx]

**VRAGENLIJST SPORTEN, BEWEGEN EN ZITTEND GEDRAG BIJ TIENERS**

We zouden je willen vragen om deze vragenlijst te beantwoorden die allerlei vragen bevat over sporten, bewegen en zittende activiteiten (vb. TV kijken, op Facebook zitten,…). Het zal ongeveer een halfuur duren.

Niemand, behalve de onderzoekers, zal je antwoorden te zien krijgen. Dus je hoeft je geen zorgen te maken dat de leerkracht, je ouders of klasgenoten je antwoorden zullen zien. Er zijn geen ‘juiste’ of ‘foute’ antwoorden. Je duidt het antwoord aan dat het meest bij jou past. De vragenlijst bestaat uit zes verschillende delen, het is de bedoeling dat deze allemaal ingevuld worden.


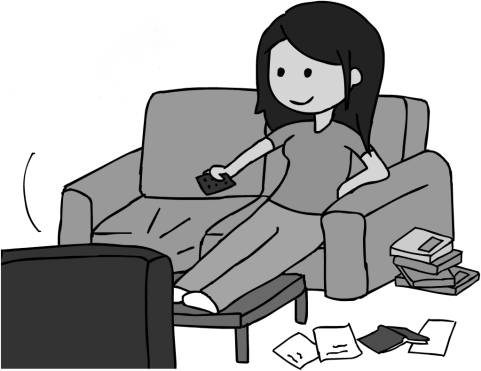
Je deelname aan deze studie is anoniem en vrijwillig. Als je deze vragenlijst dus niet wilt invullen, mag je ons dit zeggen. Als je deze vragenlijst wel invult, geef je toestemming dat wij jou gegevens mogen gebruiken voor ons project.

Alvast heel erg bedankt voor je hulp! ☺

**Deel 1: Enkele vragen over jezelf**

1. Geef hier het nummer dat je hebt gekregen van de onderzoeker.

|  |  |  |  |  |
| --- | --- | --- | --- | --- |

2. Wat is de naam van je school?

..……………………………………………....……………………………………………

3. Welke richting volg je op school?

..……………………………………………....……………………………………………

4. Wat is de datum van vandaag?

..……………………………………………

5. In welk jaar ben je geboren?

..……………………………………………

6. In welke maand valt je verjaardag?

..……………………………………………

7. Ben je een jongen of een meisje?

- Jongen
- Meisje

8. Waar ben je geboren?

- België
- In een ander land dan België: ..……………………………………………

9. Welke taal spreek je meestal thuis?

- Nederlands/Vlaams
- Frans
- Engels
- Turks
- Marokkaans
- Andere: ..……………………………………………

10. Bij wie woon je? *(Je mag meer dan 1 bolletje aanduiden.)*

- Bij mijn moeder en vader samen
- Alleen bij mijn moeder
- Alleen bij mijn vader
- Bij mijn moeder en haar nieuwe partner
- Bij mijn vader en zijn nieuwe partner
- Bij mijn grootouders
- Bij andere volwassenen

11. Wat is het hoogst behaalde diploma van je moeder?

- Lager onderwijs
- Middelbaar onderwijs
- Hoger onderwijs (niet universiteit)
- Universiteit
- Ik weet het niet.

12. Wat is het hoogst behaalde diploma van je vader ?

- Lager onderwijs
- Middelbaar onderwijs
- Hoger onderwijs (niet universiteit)
- Universiteit
- Ik weet het niet.

**Deel 2: Sport en bewegen**

1. Hoe ga je MEESTAL naar school?

- Met de fiets
- Te voet
- Met de auto, trein of bus

Hoe lang duurt het ongeveer om van jouw huis naar school te gaan? *(in minuten)*

..……………………………………………

2. Hoeveel uur lichamelijke opvoeding of sport krijg je tijdens de week op school?
*(Reken hier ook de zwemlessen bij.)*

..……………………………………………

3. Wat doe je meestal tijdens de speeltijd?

- Zitten
- Staan
- Wandelen
- Sporten
- Er is geen speeltijd.

4. Wat doe je meestal tijdens de middagpauze?

- Zitten
- Staan
- Wandelen
- Sporten
- Er is geen middagpauze.

5. Kan je sport of beweging doen op jouw school? *(buiten de lessen)*

- Ja
- Nee, op geen enkel moment **🡪 Ga meteen verder naar vraag 10 op de volgende pagina**

6. Op welke momenten kan je aan sport en beweging doen op school? *(Je mag meer dan 1 bolletje aanduiden.)*

- Tijdens de speeltijd
- Tijdens de middagpauze
- Tijdens de studie
- Na de schooluren
- Op woensdagnamiddag
- Bij klas- en schooltornooien
- Andere: ..……………………………………………

7. Neem je aan 1 van deze activiteiten deel?

- Ja
- Nee **🡪 Ga meteen verder naar vraag 10 onderaan deze pagina**

8. Aan welke activiteiten op school neem je deel? *(Je mag meer dan 1 bolletje aanduiden.)*

- Sport tijdens de speeltijd
- Sport tijdens de middag
- Sport tijdens de studie
- Sport na de schooluren
- Sport op woensdagnamiddag
- Klas- en schooltornooien
- Andere: ..……………………………………………

9. Hoeveel uur besteed je samen aan al deze sportactiviteiten op school? (Speeltijd + over de middag + na de schooluren + woensdagnamiddag + klastornooien, …)

- Een paar keer per jaar
- 1 uur per maand
- 2 uur per maand
- 3 uur per maand
- 1 uur per week
- 2 uur per week
- 3 uur per week
- 4 uur per week
- Meer dan 4 uur per week

***Bij volgende vragen moet je een gewone schoolweek voor ogen nemen!

PAS OP! De verplaatsing van en naar school en het fietsen en wandelen als sport mogen niet meegerekend worden bij vragen 10 tot 13.
Met vrije tijd wordt bijvoorbeeld bedoeld: hoe ga je naar de sportclub, je vrienden, de film,…?
Je mag slechts 1 bolletje kleuren bij deze vragen.***

10. Hoe lang wandel je meestal in je VRIJE TIJD tijdens een WEEKdag?

- Ik wandel niet.
- 5-10 minuten
- 11-20 minuten
- 21-30 minuten
- 31-40 minuten
- 41-50 minuten
- 51-60 minuten
- Meer dan één uur

11. Hoe lang fiets je meestal in je VRIJE TIJD tijdens een WEEKdag?

- Ik fiets niet.
- 5-10 minuten
- 11-20 minuten
- 21-30 minuten
- 31-40 minuten
- 41-50 minuten
- 51-60 minuten
- Meer dan één uur

12. Hoe lang wandel je meestal in je VRIJE TIJD tijdens een WEEKENDdag?

- Ik wandel niet.
- 5-10 minuten
- 11-20 minuten
- 21-30 minuten
- 31-40 minuten
- 41-50 minuten
- 51-60 minuten
- Meer dan één uur

13. Hoe lang fiets je meestal in je VRIJE TIJD tijdens een WEEKENDdag?

- Ik fiets niet.
- 5-10 minuten
- 11-20 minuten
- 21-30 minuten
- 31-40 minuten
- 41-50 minuten
- 51-60 minuten
- Meer dan één uur

14. Ga je naar een discotheek en/of fuif tijdens een normale week, van maandag tot en met zondag?

- Nee **🡪 Ga meteen verder naar vraag 17 op de volgende pagina**
- Ja

15. Hoeveel uur besteed je gemiddeld aan discotheek- en of fuifbezoek per week?

- 1 - 2 uur per week
- 3 - 4 uur per week
- 5 - 6 uur per week
- 7 - 8 uur per week
- 9 - 10 uur per week
- Meer dan 10 uur per week

16. Hoeveel uur dans je dan gemiddeld tijdens dit discotheek- en/of fuifbezoek per week?

- 0 uur per week
- 1 - 2 uur per week
- 3 - 4 uur per week
- 5 - 6 uur per week
- 7 - 8 uur per week
- 9 - 10 uur per week
- Meer dan 10 uur per week

17. Geef de 3 belangrijkste sporten die je tijdens je VRIJE TIJD het meest beoefent.
*Let op! De les lichamelijke opvoeding en de sport op school tellen niet mee!*

17.1. Mijn eerste sport

- Ik doe geen sport **🡪 Ga meteen verder naar deel 3 (pagina 9)**
- Mijn eerste sport is ..……………………………………………

Vraag A: Doe je deze sport in een club?

- Ja
- Nee

Vraag B: Doe je mee aan competitie (wedstrijden)?

- Ja
- Nee

Vraag C: Hoe vaak doe je deze sport?

- Ik doe deze sport af en toe, maar zeker niet elke week.
- 1 keer per week
- 2 keer per week
- 3 keer per week
- 4 keer per week
- 5 keer per week
- 6 keer per week
- 7 keer per week
- Meer dan 7 keer per week

Hoeveel uur per week beoefen je deze sport? *(Tel alles op vb. als je 1 uur je sport doet op dinsdag en 1 uur op donderdag, vul je hier ‘2’ in)*

..……………………………………………

17.2. Mijn tweede sport

- Ik doe geen tweede sport **🡪 Ga meteen verder naar deel 3 (pagina 9)**
- Mijn tweede sport is ………………………………………..

Vraag A: Doe je deze sport in een club?

- Ja
- Nee

Vraag B: Doe je mee aan competitie (wedstrijden)?

- Ja
- Nee

Vraag C: Hoe vaak doe je deze sport?

- Ik doe deze sport af en toe, maar zeker niet elke week.
- 1 keer per week
- 2 keer per week
- 3 keer per week
- 4 keer per week
- 5 keer per week
- 6 keer per week
- 7 keer per week
- Meer dan 7 keer per week

Hoeveel uur per week beoefen je deze sport? (*Alles optellen zoals bij ‘Mijn eerste sport’.)*

..……………………………………………

17.3. Mijn derde sport

- Ik doe geen derde sport **🡪 Ga meteen verder naar deel 3 (pagina 9)**
- Mijn derde sport is ..……………………………………………

Vraag A: Doe je deze sport in een club?

- Ja
- Nee

Vraag B: Doe je mee aan competitie (wedstrijden)?

- Ja
- Nee

Vraag C: Hoe vaak doe je deze sport?

- Ik doe deze sport af en toe, maar zeker niet elke week.
- 1 keer per week
- 2 keer per week
- 3 keer per week
- 4 keer per week
- 5 keer per week
- 6 keer per week
- 7 keer per week
- Meer dan 7 keer per week

Hoeveel uur per week beoefen je deze sport? *(Alles optellen zoals bij ‘Mijn eerste sport’.)*

..……………………………………………

**Deel 3: Zittende activiteiten tijdens de week en het weekend**

1. Hoeveel uur per dag ongeveer doe je volgende activiteiten al zittend op één **WEEKDAG?** (maandag tot en met vrijdag)

|  | Nooit | 15 min. /dag | 30 min. /dag | 1u /dag | 1,5u /dag | 2u /dag | 2,5u /dag | 3u /dag | 3,5u /dag | >4u /dag |
| --- | --- | --- | --- | --- | --- | --- | --- | --- | --- | --- |
| TV/Dvd’s kijken |  |  |  |  |  |  |  |  |  |  |
| Spelletjes spelen op computer, spelconsole of op je smartphone |  |  |  |  |  |  |  |  |  |  |
| Op Internet zitten, e-mailen, chatten, Facebook, instagram,.. op de computer of op je smartphone |  |  |  |  |  |  |  |  |  |  |
| Huiswerk maken |  |  |  |  |  |  |  |  |  |  |
| In de auto, bus, trein of op een brommer zitten |  |  |  |  |  |  |  |  |  |  |
| Een boek of tijdschrift lezen |  |  |  |  |  |  |  |  |  |  |
| Zittende hobby’s vb. tekenen of naar muziek luisteren |  |  |  |  |  |  |  |  |  |  |
| Al zittend met vrienden of vriendinnen praten, iets gaan drinken |  |  |  |  |  |  |  |  |  |  |

1. Hoeveel uur per dag ongeveer doe je volgende activiteiten al zittend op één **WEEKENDDAG**? (zaterdag of zondag)

|  | Nooit | 15 min. /dag | 30 min. /dag | 1u /dag | 1,5u /dag | 2u /dag | 2,5u /dag | 3u /dag | 3,5u /dag | >4u /dag |
| --- | --- | --- | --- | --- | --- | --- | --- | --- | --- | --- |
| TV/Dvd’s kijken |  |  |  |  |  |  |  |  |  |  |
| Spelletjes spelen op computer, spelconsole of op je smartphone |  |  |  |  |  |  |  |  |  |  |
| Op Internet zitten, e-mailen, chatten, Facebook, instagram,.. op de computer of op je smartphone |  |  |  |  |  |  |  |  |  |  |
| Huiswerk maken |  |  |  |  |  |  |  |  |  |  |
| In de auto, bus, trein of op een brommer zitten |  |  |  |  |  |  |  |  |  |  |
| Een boek of tijdschrift lezen |  |  |  |  |  |  |  |  |  |  |
| Zittende hobby’s vb. tekenen of naar muziek luisteren |  |  |  |  |  |  |  |  |  |  |
| Al zittend met vrienden of vriendinnen praten, iets gaan drinken |  |  |  |  |  |  |  |  |  |  |

**Deel 4: Onderbreken van zit-tijd**

**Met 'het onderbreken van de tijd die je al zittend doorbrengt', bedoelen we rechtstaan, stretchen of een beetje rondwandelen tijdens het doen van een activiteit die je normaal al zittend doet. Tel enkel de onderbrekingen mee die je doet omdat je bewust de tijd die je al zittend doorbrengt wil onderbreken - tel niet de onderbrekingen mee zoals naar het toilet gaan of iets halen om te eten.**

1. Als je 1 uur naar TV/Dvd’s kijkt, hoe vaak sta je dan normaal gezien recht, stretch je of wandel je een beetje rond?

- Nooit
- 1 keer
- 2 keer
- 3 keer
- 4 keer of meer
- Ik kijk nooit een volledig uur naar TV/Dvd’s.

2. Als je 1 uur een computer/spelconsole/smartphone gebruikt, hoe vaak sta je dan normaal gezien recht, stretch je of wandel je een beetje rond?

- Nooit
- 1 keer
- 2 keer
- 3 keer
- 4 keer of meer
- Ik gebruik nooit een volledig uur een computer/spelconsole/smartphone.

3. Tijdens een normale zittende les op school, hoe vaak sta je recht, stretch je of wandel je een beetje rond?

- Nooit
- 1 keer
- 2 keer
- 3 keer
- 4 keer of meer

4. Hoe belangrijk is het voor de gezondheid volgens jou om af en toe eens recht te staan, te stretchen of rond te wandelen als je een lange tijd neerzit?

- Heel belangrijk
- Een beetje belangrijk
- Soms belangrijk, soms niet belangrijk
- Niet belangrijk
- Helemaal niet belangrijk

**Deel 5: Enkele vragen over jou, je ouders en vrienden**

1. Wat vind je van volgende activiteiten?

|  | Heel leuk | Een beetje leuk | Soms leuk/ soms niet leuk | Niet leuk | Helemaal niet leuk |
| --- | --- | --- | --- | --- | --- |
| Wat vind je van sporten en bewegen? |  |  |  |  |  |
| Wat vind je van TV/Dvd’s kijken? |  |  |  |  |  |
| Wat vind je van spelletjes spelen op de computer, spelconsole of smartphone? |  |  |  |  |  |
| Wat vind je van op Internet zitten op de computer of smartphone (vb. e-mailen, chatten, Facebook, Instagram,…)? |  |  |  |  |  |

2. We zouden graag weten of je het eens bent met volgende uitspraken.
*(Duid telkens één antwoord aan, ook als je niet aan sport doet)*

|  | Helemaal mee eens | Een beetje mee eens | Soms niet/ soms wel mee eens | Een beetje niet mee eens | Helemaal niet mee eens |
| --- | --- | --- | --- | --- | --- |
| Ik ben zeker dat ik zal sporten en bewegen, ook als ik vroeg moet opstaan. |  |  |  |  |  |
| Ik ben zeker dat ik zal sporten en bewegen, ook als mijn vrienden iets anders willen doen. |  |  |  |  |  |
| Ik ben zeker dat ik zal sporten en bewegen, ook als ik nog veel werk heb voor school. |  |  |  |  |  |
| Ik ben zeker dat ik zal sporten en bewegen, ook als het lastig of moeilijk is. |  |  |  |  |  |

3. We zouden graag weten of je het eens bent met volgende uitspraken.
*(Duid telkens één antwoord aan, ook als je niet aan sport doet)*

|  | Helemaal mee eens | Een beetje mee eens | Soms niet/ soms wel mee eens | Een beetje niet mee eens | Helemaal niet mee eens |
| --- | --- | --- | --- | --- | --- |
| Ik vind sport en bewegen goed omdat mijn conditie en mijn gezondheid beter worden |  |  |  |  |  |
| Ik vind sport en bewegen goed omdat ik met vrienden samen ben of nieuwe mensen leer kennen. |  |  |  |  |  |
|  | Helemaal mee eens | Een beetje mee eens | Soms niet/ soms wel mee eens | Een beetje niet mee eens | Helemaal niet mee eens |
| Ik vind sport en bewegen goed omdat ik er plezier aan beleef. |  |  |  |  |  |
| Ik vind sport en bewegen goed omdat ik tijdens het sporten of bewegen kan tonen dat ik beter ben dan anderen. |  |  |  |  |  |
| Ik vind sport en bewegen goed omdat ik me niet verveel als ik sport of beweeg. |  |  |  |  |  |
| Ik vind sport en bewegen goed omdat ik gewicht verlies en dat mijn lichaam mooier wordt. |  |  |  |  |  |

4. We zouden graag weten of je het eens bent met volgende uitspraken.
*(Duid telkens één antwoord aan, ook als je veel aan sport doet)*

|  | Helemaal mee eens | Een beetje mee eens | Soms niet/ soms wel mee eens | Een beetje niet mee eens | Helemaal niet mee eens |
| --- | --- | --- | --- | --- | --- |
| Ik kan niet veel aan sport en bewegen doen omdat ik niet genoeg tijd heb of omdat ik te veel huiswerk heb. |  |  |  |  |  |
| Ik kan niet veel aan sport en bewegen doen omdat ik het niet graag doe. |  |  |  |  |  |
| Ik kan niet veel aan sport en bewegen doen ik het niet goed kan. |  |  |  |  |  |
| Ik kan niet veel aan sport en bewegen doen omdat ik niet altijd vervoer heb om er te geraken. |  |  |  |  |  |
| Ik kan niet veel aan sport en bewegen doen omdat ik niet mag van mijn ouders/verzorgers. |  |  |  |  |  |

5. Hoe vaak sporten of bewegen je vrienden?

- Nooit
- Bijna nooit
- Soms
- Vaak
- Heel vaak

6. Hoe vaak sporten of bewegen je ouders/verzorgers?

- Nooit
- Bijna nooit
- Soms
- Vaak
- Heel vaak

7. Hoe vaak sporten of bewegen je vrienden **samen met jou**?

- Nooit
- Bijna nooit
- Soms
- Vaak
- Heel vaak

8. Hoe vaak sporten of bewegen je ouders/verzorgers **samen met jou**?

- Nooit
- Bijna nooit
- Soms
- Vaak
- Heel vaak

9. Hoe vaak moedigen je vrienden je aan om te sporten of bewegen?

- Nooit
- Bijna nooit
- Soms
- Vaak
- Heel vaak

10. Hoe vaak moedigen je ouders/verzorgers je aan om te sporten of bewegen?

- Nooit
- Bijna nooit
- Soms
- Vaak
- Heel vaak

11. Deze vraag gaat over hoe vaak je vrienden zittende activiteiten doen.

|  | Nooit | Bijna nooit | Soms | Vaak | Heel vaak |
| --- | --- | --- | --- | --- | --- |
| Hoe vaak kijken je vrienden TV/Dvd’s? |  |  |  |  |  |
| Hoe vaak spelen je vrienden spelletjes op de computer, spelconsole of smartphone? |  |  |  |  |  |
| Hoe vaak zitten je vrienden op Internet (vb. e-mailen, chatten, Facebook, Instagram,…) op de computer of smartphone? |  |  |  |  |  |

12. Deze vraag gaat over hoe vaak je ouders of verzorgers zittende activiteiten doen.

|  | Nooit | Bijna nooit | Soms | Vaak | Heel vaak |
| --- | --- | --- | --- | --- | --- |
| Hoe vaak kijken je ouders/verzorgers TV/Dvd’s? |  |  |  |  |  |
| Hoe vaak spelen je ouders/verzorgers spelletjes op de computer, spelconsole of smartphone? |  |  |  |  |  |
| Hoe vaak zitten je ouders/verzorgers op Internet (vb. e-mailen, chatten, Facebook, Instagram,…) op de computer of smartphone? |  |  |  |  |  |

13. Deze vraag gaat over hoe vaak je vrienden zittende activiteiten doen **samen met jou.**

|  | Nooit | Bijna nooit | Soms | Vaak | Heel vaak |
| --- | --- | --- | --- | --- | --- |
| Hoe vaak kijken je vrienden TV/Dvd’s samen met jou? |  |  |  |  |  |
| Hoe vaak spelen je vrienden spelletjes op de computer, spelconsole of smartphone samen met jou? |  |  |  |  |  |
| Hoe vaak zitten je vrienden op Internet (vb. e-mailen, chatten, Facebook, Instagram,…) op de computer of smartphone samen met jou? |  |  |  |  |  |

14. Deze vraag gaat over hoe vaak je ouders of verzorgers zittende activiteiten doen **samen met jou.**

|  | Nooit | Bijna nooit | Soms | Vaak | Heel vaak |
| --- | --- | --- | --- | --- | --- |
| Hoe vaak kijken je ouders/verzorgers TV/Dvd’s samen met jou? |  |  |  |  |  |
| Hoe vaak spelen je ouders/verzorgers spelletjes op de computer, spelconsole of smartphone samen met jou? |  |  |  |  |  |
| Hoe vaak zitten je ouders/verzorgers op Internet (vb. e-mailen, chatten, Facebook, Instagram,…) op de computer of smartphone samen met jou? |  |  |  |  |  |

15. Deze vraag gaat over hoe vaak je vrienden je aanmoedigen om minder zittende activiteiten te doen.

|  | Nooit | Bijna nooit | Soms | Vaak | Heel vaak |
| --- | --- | --- | --- | --- | --- |
| Hoe vaak moedigen je vrienden je aan om minder TV/Dvd’s te kijken? |  |  |  |  |  |
| Hoe vaak moedigen je vrienden je aan om minder spelletjes te spelen op de computer, spelconsole of smartphone? |  |  |  |  |  |
| Hoe vaak moedigen je vrienden je aan om minder op Internet te zitten (vb. e-mailen, chatten, Facebook, Instagram,…) op de computer of smartphone? |  |  |  |  |  |

16. Deze vraag gaat over hoe vaak je ouders of verzorgers je aanmoedigen om minder zittende activiteiten te doen.

|  | Nooit | Bijna nooit | Soms | Vaak | Heel vaak |
| --- | --- | --- | --- | --- | --- |
| Hoe vaak moedigen je ouders/verzorgers je aan om minder TV/Dvd’s te kijken? |  |  |  |  |  |
| Hoe vaak moedigen je ouders/verzorgers je aan om minder spelletjes te spelen op de computer, spelconsole of smartphone? |  |  |  |  |  |
| Hoe vaak moedigen je ouders/verzorgers je aan om minder op Internet te zitten (vb. e-mailen, chatten, Facebook, Instagram,…) op de computer of smartphone? |  |  |  |  |  |

**Deel 6: Nog enkele vragen over je school**

1. Hier volgen enkele uitspraken over jouw klasgenoten en jouw school. In welke mate ben jij het hiermee eens?

|  | Helemaal mee eens | Een beetje mee eens | Soms niet/ soms wel mee eens | Een beetje niet mee eens | Helemaal niet mee eens |
| --- | --- | --- | --- | --- | --- |
| Mijn klasgenoten zijn graag samen. |  |  |  |  |  |
| De meeste leerlingen van mijn klas zijn vriendelijk en behulpzaam. |  |  |  |  |  |
| Mijn klasgenoten aanvaarden me zoals ik ben. |  |  |  |  |  |
| De leerlingen in onze school zijn betrokken bij het organiseren van schoolactiviteiten. |  |  |  |  |  |
| Mijn klasgenoten en ik hebben een goede band met de leraren op school. |  |  |  |  |  |

2. Hoe vaak praat je met je ouders/verzorgers over wat je op school doet?

- Ongeveer elke dag
- Ongeveer 1 of 2 keer per week
- Ongeveer 1 of 2 keer per maand
- Minder dan 1 keer per maand
- Nooit

3. Wat denk je momenteel over school?

- Ik vind het er leuk./ Ik doe het zeer graag.
- Ik vind het redelijk leuk.
- Ik hou er niet zo erg van.
- Ik heb er een hekel aan./ Ik doe het helemaal niet graag.


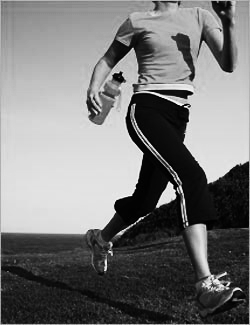


**Heel erg bedankt om mee te werken!!**
